# Malaria control in rural Malawi: implementing peer health education for behaviour change

**DOI:** 10.1186/s12992-017-0309-6

**Published:** 2017-11-20

**Authors:** Tumaini Malenga, Alinune Nathanael Kabaghe, Lucinda Manda-Taylor, Asante Kadama, Robert S. McCann, Kamija Samuel Phiri, Michèle van Vugt, Henk van den Berg

**Affiliations:** 1 Department of Health Systems and Policy Development, University of Malawi College of Medicine, Blantyre, Malawi; 2 Training and Research Unit of Excellence, University of Malawi College of Medicine, Blantyre, Malawi; 3 Academic Medical Centre, University of Amsterdam, Amsterdam, The Netherlands; 4 School of Public Health and Family Medicine, University of Malawi College of Medicine, Blantyre, Malawi; 5 Wageningen University and Research, Wageningen, The Netherlands

**Keywords:** Malaria, Health education, Implementation, Health animator, Community workshops, Behaviour change

## Abstract

**Background:**

Interventions to reduce malaria burden are effective if communities use them appropriately and consistently. Several tools have been suggested to promote uptake and use of malaria control interventions. Community workshops on malaria, using the ‘Health Animator’ approach, are a potential behaviour change strategy for malaria control. The strategy aims to influence a change in mind-set of vulnerable populations to encourage self-reliance, using community volunteers known as Health Animators. The aim of the paper is to describe the process of implementing community workshops on malaria by Health Animators to improve uptake and use of malaria control interventions in rural Malawi.

**Methods:**

This is a descriptive study reporting feasibility, acceptability, appropriateness and fidelity of using Health Animator-led community workshops for malaria control. Quantitative data were collected from self-reporting and researcher evaluation forms. Qualitative assessments were done with Health Animators, using three focus groups (October–December 2015) and seven in-depth interviews (October 2016–February 2017).

**Results:**

Seventy seven health Animators were trained from 62 villages. A total of 2704 workshops were conducted, with consistent attendance from January 2015 to June 2017, representing 10–17% of the population. Attendance was affected by social responsibilities and activities, relationship of the village leaders and their community and involvement of Community Health Workers. Active discussion and participation were reported as main strengths of the workshops. Health Animators personally benefited from the mind-set change and were proactive peer influencers in the community. Although the information was comprehended and accepted, availability of adequate health services was a challenge for maintenance of behaviour change.

**Conclusion:**

Community workshops on malaria are a potential tool for influencing a positive change in behaviour towards malaria, and applicable for other health problems in rural African communities. Social structures of influence and power dynamics affect community response. There is need for systematic monitoring of community workshops to ensure implementation fidelity and strengthening health systems to ensure sustainability of health behaviour change.

## Background

Interventions to reduce malaria burden are effective if communities use them appropriately and consistently. The use of insecticide-treated bed nets (ITN), and treating malaria with artemisinin-based combination therapy (ACT) has played a major role in averting an estimated 663 million clinical cases between 2005 and 2015 in sub Saharan Africa [1]. Despite the effectiveness of these interventions, the burden of malaria in poor, rural African communities remains high [2]. A combination of poor health systems, limited access to malaria prevention, diagnosis and treatment services, poverty, and low levels of education, hinder efforts to reduce malaria in most rural African communities [3, 4].

Although prompt and effective treatment of malaria reduces transmission, factors such as traditional beliefs, have been shown to contribute to delays in seeking care for fever in Malawi [5]. Care-seeking for fever in children under 5 years old was 58.8% in the 2014 Malawi Malaria Indicator Survey (MMIS) [6] and 67% in the 2015/16 Demographic and Health Survey (DHS) [7]. The 2014 MMIS also reported that only 52% of the population used ITNs the previous night, although ownership of at least one ITN per household had increased from 58% in 2010 to 70% in 2014 [6]. These low rates of uptake undermine the effectiveness of the interventions, and highlight the need to address factors hindering behavioural changes consistent with malaria prevention.

Several approaches, such as Information Education and Communication (IEC), Communication-for- Behavioural- Impact (COMBI), Behaviour Change Communication (BCC) and school-based health education, have been suggested and implemented to promote uptake of malaria interventions [8–12]. These approaches are aimed at enabling people to improve their health, access appropriate treatment, and adopt preventive measures. Most of these approaches however suffer from limitations; they typically assume a high level of quality of existing structures (e.g. schools), access to media (radio, television or newspaper), and in some cases, the ability to read. Moreover, some are implemented as ‘campaigns’, conducted once in the community, and with uncertain persisting effect [13].

**Fig. 1:**
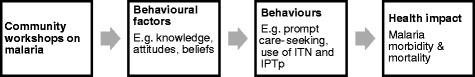
Pathway for reducing malaria burden by targeting behavioural factors. Introducing community workshops on malaria will improve knowledge of the disease and promote positive attitudes and beliefs towards malaria prevention and treatment. This would lead to the adoption of desired behaviour for seeking care or preventing disease. In the longer term and with consistency with sustaining desired health seeking behaviour, the rates of morbidity and mortality would decrease

The Majete Malaria Project (MMP) is using a Health Animator (HA) approach as a community engagement strategy to encourage health behaviour change for malaria control (Fig. 1). The HA approach of community engagement for disease control was pioneered by The Hunger Project (THP) for HIV awareness and prevention in Malawi. The strategy aims to influence a change in mind-set of vulnerable populations to encourage self-reliance. This mind-set change is achieved through community mobilisation for internalising self-reliance to overcome livelihood challenges [14]. The process involves leadership training for HAs; HAs become peer influencer and unite and commit the community to own one vision of change through collective action. The HAs engage with the community through regular community workshops, held over a period of several years. The workshops are used to educate and discuss with the community specific challenges affecting them. In addition to the malaria workshops, THP have implemented community workshops on other developmental aspects in the MMP catchment area, as part of their ‘epicentre’ strategy [15]. The ‘epicentre’ strategy aims to educate and mobilise the community towards poverty reduction through a broad set of programmes, including improved food and financial security, adult literacy and HIV prevention.

This paper evaluates the implementation of community workshops on malaria to improve use of malaria interventions in a rural setting. The paper uses the “Taxonomy of implementation outcomes” by Proctor et al. [16] to report feasibility, acceptability, appropriateness, and fidelity of community workshops on malaria.

## Methods

### Study design

This was a descriptive study, which used qualitative and quantitative methods to evaluate implementation of community workshops on malaria (henceforth “workshops” unless otherwise specified) in Chikhwawa district, southern Malawi.

### Study setting

MMP is based in communities surrounding Majete Wildlife Reserve (MWR). The reserve covers an area of approximately 700 km^2^ in Chikhwawa. *Plasmodium falciparum* malaria is endemic in Chikhwawa, with transmission throughout the year, peaking in the wet season (December to May).

The catchment area for MMP consists of three focal areas (A, B and C) with a total population of 24,153. The focal areas were selected from existing epicentres established by THP, spaced roughly evenly around the wildlife reserve to cover a total population of approximately 25,000 people, and described by McCann et al. [17]. An epicentre is a cluster of villages whose communities are mobilised to participate as a unit in THP programmes*.*

Communities in MWR perimeter rely on subsistence and small-scale rain-fed crop and livestock farming. Reported literacy levels for Chikhwawa district in 2010 were 73.8 and 54.9% for men and women, respectively [18]. Due to its remoteness, the MMP catchment area where the workshops were implemented is likely to have even lower literacy levels than the district average.

### Implementation outcomes

‘Feasibility’ is the degree of success of implementing an intervention within a specified setting; the extent to which the intervention can be practically implemented [16]. In practice, components such as human, financial and material resources, are required for implementation of an intervention [19]. In this study, feasibility of implementing community workshops on malaria is measured from HA community workshop records, and reported from qualitative interviews by the HAs.

‘Appropriateness’ examines how proper and how well the intervention fits within a specific setting [16]; here, how the intervention interacts with the community in a cultural context is key for uptake by both provider and beneficiary [19]. Appropriateness is reported from the qualitative interviews with the HAs.

‘Acceptability’ measures satisfaction with a specific intervention of both provider and recipient [16]. Acceptability is reported from qualitative interviews and attendance records.

‘Fidelity’ is the extent to which an intervention was implemented as intended, planned, and designed [16]. In this study, fidelity was measured from standard peer-assessment and MMP-evaluation forms.

### Health animators (HAs)

HAs are a cadre of community-based volunteers tasked with implementing community workshops. The minimum requirements for HAs are: ability to read and write; willingness and availability to conduct the workshops without financial gain; and permanent residence within the catchment community. There are no gender restrictions and any person above the age of 18 was eligible to be an HA. HAs were identified and approached by village leaders, and trained by MMP to educate their community about malaria. On average, each village had 1 HA, with larger villages having more than 1 HA and some smaller villages sharing one animator. Although HAs are volunteers, each HA was given a bicycle to use for transportation when attending coordination meetings or for conducting workshops. They were also given branded T-shirts and caps for easy identification. In addition, during their trainings, they received a standard upkeep cash allowance. There were no other incentives provided.

### Malaria manual and training

The MMP together with Chikhwawa District Health Office, African Parks and THP personnel developed a malaria manual for HAs which was translated into Chichewa, the local language. The manual (which will be published separately) has 24 learning units prepared for a series of workshops to be completed in an annual cycle. Each learning unit is presented during each workshop; the learning units include topics such as malaria transmission, diagnosis and treatment, vector behaviour, vector ecology, bed nets, community activities, risk factors, the health system, and a participatory evaluation. Each learning unit is structured with learning objectives, main topic and group work. The main topics are interactive, with questions from facilitator and responses from participants. Group work requires a discussion in groups of 10–15 people per workshop on the topic, and its application in the community. Complex information is illustrated using simplified diagrams which are printed on A3 paper.

All HAs initially underwent a two-day introduction on the Vision, Commitment and Action workshop concept (VCA). The concept involves creating a vision for the future of the community, committing to achieving the vision and formulating a specific action plan to achieve the vision. All HAs then underwent a five-day training course based on the malaria manual. The course was conducted in Chichewa, and facilitated by both THP and MMP staff. It was designed to be interactive with trainees (i.e. HAs) being asked their beliefs and attitudes concerning the topic before discussing it. The HAs also performed and practiced malaria-themed plays, songs or poems in preparation of the workshops. At the end of the training, each HA received a malaria manual and a set of the A3 illustrative posters to use in their respective villages. Government-employed Community Health Workers, known as Health Surveillance Assistants (HSA), also attended the training. The HA training was based on the malaria manual and modelled the workshops. HAs had annual refresher training.

### Community workshop implementation

The community workshops on malaria were implemented in all villages within the three focal areas. After the HA training, MMP briefed all village chiefs about the workshops, emphasising the need for their support and involvement. The HAs conducted village workshops in their respective villages, with the intention of having a workshop every fortnight, using the same approach as the training. Before each workshop, the HA informed the village chief. The village chief sent a message in the village a day prior to and on the day of the workshop. The HA also notified the HSA and village health committee to attend the workshops.

A workshop was facilitated by 1 HA; two other people, often other HAs, assisted. If two additional animators were not available, then a community member attending the workshops was chosen. Each workshop included a short play, poem or song with a malaria message, depending on the HAs available for facilitation, at the beginning. The workshops were planned to take 1 hour and 45 min although some workshops were shorter than this.

### Monitoring and evaluation of community workshops

During the workshops, one individual served as a rapporteur responsible for evaluating the workshop using a standard evaluation form. He/she recorded observed strengths and weaknesses and the other acted as a secretary, recording the date of workshop, topic discussed, and attendance (by name and gender). Also, the number of suspected, self-reported malaria cases among the households of workshop participants since the previous workshop was recorded. The rapporteur and secretary reports were used to monitor attendance and identify implementation challenges. Each question in the self-reported evaluation form was graded as follows: 1 = not done; 2 = done. In addition to the evaluation form, the reporting forms contained open questions asking about strengths of the workshop, aspects needing improvement, issues or concerns the participants provide as feedback, and problems in preparing (and conducting) the workshops.

A coordination meeting for HAs and community health workers was scheduled within each focal area, at monthly or less frequent intervals. During these meetings, summary reports on the attendance and self-reported assessment were shared and major challenges faced were documented and potential ways of improvement were discussed.

The implementation fidelity of the workshops was independently assessed by MMP personnel in four randomly selected workshops (two for each focal area B and C). The assessment used a standard graded evaluation form and recorded strengths and weaknesses of the workshop. Each question on the form was graded as follows: 1 = not done; 2 = partially done; 3 = not done.

### Qualitative assessment

Three focus group discussions (FGD) with 8–12 HAs were conducted from October to December 2015. Seven in-depth interviews (IDI) with HAs were also conducted between October 2016 and February 2017. The majority of the HAs had primary-level education, were married and were subsistence farmers. All FGDs and IDIs were conducted in Chichewa. Ethical approval for interviews was sought from and given by the University of Malawi’s College of Medicine Research Ethics Committee (certificate number P05/15/1724). Participants were requested to provide written consent in the presence of a witness prior to the commencement of the interview. All interviews were digitally recorded. A semi-structured interview guide was used to facilitate the conversation, focusing on the experiences with training on health animation, conducting workshops in their respective villages, successes and challenges of the workshops.

### Data management and analysis

Self-reported and attendance forms were entered into a spreadsheet and the data summarised using Microsoft Excel. *P*-values were calculated using Stata software version 13. Digital recordings of the qualitative interviews were transcribed verbatim, translated into English, and coded using QSR International NVivo 10 software. The English IDI transcripts were independently coded by two individuals to identify emerging themes in the experiences. FGDs were coded and analysed by one researcher.

## Results

### Health animators

Table 1 summarises the total number of HAs trained, villages and populations of the three focal areas. In total, two trainings, one for focal area A and one combined focal area B and C were funded and conducted by MMP; 77 HAs from all focal areas completed the five-day malaria HA training. Between 33% and 48% of the HAs were female. During the three annual cycles of implementing the workshops, seven animators were replaced: two underperformed (were not conducting workshops) and five relocated from their villages. Replacement HAs underwent a two-day orientation with MMP staff for the malaria manual and were provided additional on-the-job training by their fellow HAs.

**Table 1:** Population and number of Health Animators trained

Item	Focal area			
	A	B	C	All
Population size (2014)	4131	8444	11,578	24,153
Number of villages	21	11	30	62
Number of households	857	2139	2400	5396
Persons per household	4.8	3.9	4.8	4.5
Number of Health Animators (% female)	23 (48%)	24 (33%)	30 (40%)	77 (40%)
Households per Health Animator	37.2	89.1	80	70.1
Number of community workshop sites*	21	12	21	54
Population per community workshop site**	197	704	551	447

*Some community workshop sites combined more than one village; in one instance, a village hosted two sites; **This is the average expected number of people per workshop and is derived from dividing population size by the number of workshop sites

From the qualitative interviews, some of the animators mentioned that they already had some understanding about malaria before the introduction of the programme and were making use of bed nets and adopting desirable behaviour for malaria prevention. Other animators believed malaria could not be controlled in their community prior to their training, similar to the community beliefs.

*“This project has really helped me. Before I started working for the project I had ignorant thinking about malaria, I was fond of using medicine at home. Even when my wife was sick I would not take her to the hospital immediately. I would rush to buy Brufen® (ibuprofen) and Panadol® (paracetamol), and I would give it to her. When her fever dropped a little I would think she had recovered. Within three days she’d start up again. So, I really suffered with this. Even a child… if the mother suffered from malaria, the child would be next. There was chaos in my household.” HA (FGD 010 HA).*

Equipped with a change of mind-set and new knowledge on the cause of malaria and prevention methods, they made sure to lead by example through the choices made in their own home to influence their peers into taking their lessons seriously.

*“I was among the people who did not believe malaria could be controlled, even at the time we were being taught, but now I believe it is possible” HA (IDI 006 HA).*

### Attendance of village workshops

The total number of community workshops from January 2015 to June 2017 in all focal areas was 2704. From a sample of 172 workshops conducted, each workshop was conducted by three HAs on average (Table 2). The average attendance of workshops was 45 persons per workshop; focal area B had the highest attendance (75.5 per workshop) among the three focal areas; attendance appeared to be constant over the 3 years. Reported female attendance was 56% and 66% in focal areas A and C, respectively. All focal areas reported attendance of the village chief in 100% of the workshops. Attendance of HSA varied by focal area and year; focal area B and 2015, the first year of implementation, had the highest reported HSA attendance while focal area A and the year 2017 had the lowest. About 90% of the workshops on average had a village health committee member in attendance. The village health committee is an intermediary between the community and the health system, presenting and discussing health related concerns from the community with health facility personnel and vice versa.

**Table 2:** Total number of village workshops and attendance

Item	Focal area			Year			
	A	B	C	2015	2016	2017*	All
Annual average number of community workshops completed per site**	24	23	24	24.7	22.7	15.7	
Number of community workshops for which records are available (n)	86	44	42	39	52	81	172
Number of Health Animators present per community workshop	3.1	4.1	2.7	4.1	3.0	3.0	3.3
Participants per community workshop	34.0	75.7	54.3	46.4	45.3	45.9	45.8
Average % of female participants	56%		66%				
Average % of population participating per community workshop	17%	11%	10%	10%	10%	10%	10%
Community workshops with village chief present (%)	100%	100%	100%	100%	100%	100%	100%
Community workshops with HSA present (%)	22%	66%	26%	62%	46%	14%	34%
Community workshops with health committee member present (%)	88%	93%	88%	92%	88%	89%	90%

*All meetings up to July 2017; ** average per focal area meetings exclude 2017 counts. *HSA* Health Surveillance Assistant

According to the qualitative interviews of HAs, the workshops were attended by men, women and the youth. Women were the highest number attending. Occasionally men and the youth would attend. Some of the reasons for low male attendance and participation were: (i) working to earn a living for the household, (ii) other activities occurring in the village, and (iii) a belief that health information and activities are the responsibility of the woman in the household.

*“Sometimes we have only women attending, say fifteen… At other times with ten attendees, only two men… As you know, at the moment there is a problem of famine, and although we get handouts, people rush for piece work in the gardens...” HA (IDI 002 HA).*

*“Other times very few people came, especially if the meetings coincided with other activities… For example if we held a meeting on the day there was a football match, that meant that we shared the crowd and we would have fewer people attending,” (IDI 005 HA).*

*“Men do not attend these meetings in large numbers. It is mostly women. Because the woman is the one that witnesses the illness of her children most often” HA (IDI 004 HA).*

The qualitative interviews suggested that the presence and reputation of a village chief had a strong influence on the numbers of people attending. The authority of the chief would be used to advertise and invite people to attend the meetings and if the chief was well respected, attendance would be high.

*“To call for a meeting we need to use the chief, without announcing through the chief people would not come to our meeting” (IDI 002 HA).*

At times, when the attendance was declining, the HAs sought permission to utilise meetings already organised by the chief to present a short workshop to the audience available.

*“The method we are using now is that when a chief calls for his own village meeting we take this opportunity to ask if we can use his meeting to relay our [malaria] message. This is because now, when the chief calls for our meetings the people would say ‘oh that [meeting] is useless, the project has no benefits’, they would say that even though we know the benefits of the project” (FGD 001 HA).*

In focal area B, where HSAs most frequently attended, the number of participants was also high (Table 2). The HAs reported that collaborating with staff from their local health centres helped validate their work to the community. They would at times run workshops with the HSA when they needed help with giving information in a part of the manual where they did not feel confident.

*“We did work with the health centre staff. They would help us at times when we forget the sentence, or to confirm an important message” (FGD 001 HA).*

A temporary decline in attendance was also attributed to the lack of incentives for people to invest time in attending.

*“When we started running the workshops things were working well. But at the moment there are a few things that have put people off [attending meetings]. As animators, we try to work well with the community but there are things that they had been promised they would be given; things [bed nets] to protect them from malaria. They held on to that promise. When we have meetings we do not have the high attendance like we did when we started” (FGD 001 HA).*

### Community engagement and malaria workshops

According to the responses to open questions on the self-reporting forms, there was active interaction and involvement of the participants in the workshops (Table 3); 40% and 20% of the workshops reported as strength of the workshop that participants asked and/or answered questions, and participated actively, respectively. Areas such as *drama, songs or poems* (29%), preparation of the topic by the HA, interacting more with participants and the time of starting the workshops, needed to improve. Workshop attendants were concerned with the inadequacy of health services and lack of bed nets.

**Table 3:** Self evaluation responses

Year	2015	2016	2017	All
	(*n* = 39)	(*n* = 52)	(*n* = 81)	(*n* = 172)
Strengths				
Participants able to ask and/or answer questions	31%	38%	46%	40%
Active participation	38%	12%	16%	20%
Participants able to follow/understand the topic	21%	13%	11%	14%
Time management	3%	4%	6%	5%
Participants able to give comments	0%	4%	5%	3%
Areas needing improvement				
Conducting drama, songs or poems	21%	25%	36%	29%
People to come on time	13%	27%	15%	18%
None needed	0%	10%	19%	12%
Encourage people to come to the community workshops	21%	13%	4%	10%
Animator needs to prepare for the day’s topic	18%	4%	9%	9%
Timely inform village chief about next community workshop	10%	2%	0%	3%
Community concerns				
Inadequate health services nearby	13%	23%	17%	18%
Insufficient bed nets received	21%	6%	19%	15%
Delayed distribution of bed nets	49%	4%	0%	12%
Need for house improvement and/or larval source management	3%	10%	10%	8%
Need to continue the community workshops	0%	4%	5%	3%
Implementation problems				
None reported	0%	25%	35%	24%
Rains (affecting attendance)	31%	21%	11%	19%
Funerals (affecting attendance)	5%	23%	14%	15%
Low attendance	26%	2%	6%	9%
Sickness (affecting attendance)	3%	12%	9%	8%
Inadequate materials	0%	6%	6%	5%

The qualitative interviews suggested that malaria was considered the biggest threat to health in all three catchment areas. This was the main motivation for the initial commitment with attending meetings as the villagers were seeking to discover alternatives to managing the disease in their homes. The workshops were first met with scepticism, due to the novelty of information delivery method. The community perception of the possibility of existing without malaria was preventing them from accepting the information in the workshops at the start of the programme.

*“The people did not believe it was possible to exist without malaria because this was a disease that [even] their ancestors had left behind” (FGD 002 HA).*

Some people in the community believed that malaria originated from sources other than a mosquito bite.

*“The people thought malaria is caused by walking long distances, or maybe eating [too much] sugarcane…these beliefs come from parents, these are habits that we are born into” HA (IDI 002 HA).*

There was variation in the time it took for participants to comprehend the message in the lessons.

*“People did not know what malaria was and what caused it…that was before they were given any information on malaria [through the project]” HA (IDI 006 HA).*

Some people understood and took the advice immediately. Others required repeated lessons to understand the importance of preventing illness and seeking appropriate treatment with modern medicine.

*“We would tell them to just try to adhere to advice from the hospital. They should take all the medication prescribed and complete the dose to test if it really helps. Then they can decide for themselves whether the interventions work. So slowly people would understand” HA (IDI 002 HA).*

According to the HAs, the measure of understanding was based on the frequent interaction with the crowd. People asking questions signified a level of critical thought. It showed that they understood better when things were explained more elaborately and their questions were addressed.

*They [workshop participants] used to take an active role, and that was pleasing… It meant that they understood… They participated, they asked and answered questions appropriately. So we knew that people were pleased and understood what we were doing” (IDI 005 HA).*

Through explaining the causal pathway and the role of an intervention in ending the life cycle of malaria, it seemed that people were better able to understand the use and importance of malaria control interventions.

*“…because we explained how important mosquitoes are with malaria transmission. So they appreciated the protection from sleeping under a net…” (IDI 005 HA).*

The attendance increased and the sessions became more interactive with people freely asking questions and appearing to comprehend the message.

The HAs mentioned in the interviews that they advised the participants to test the lessons learned; sleeping under a bed net and seeking treatment early. The most frequently reported change noted by the HAs was the adoption and practice of seeking treatment early when fever develops.

*“In the beginning people would just take pain killers when they had a fever…now they know and understand the importance of going to the hospital to test for malaria” (FGD 002 HA).*

Self-medicating with painkillers and visiting traditional healers were a common practice because of how easily accessible and convenient they were in the community.

In some instances, the message promoted by the animator was not complemented well with the service provision of the health systems in their communities. There were times the advice the animator was giving to the community was not supported with standard practice in the health centre. This difference was evident with the promotion of testing for malaria to confirm infection. Some health centres did not have a supply of malaria rapid diagnostics tests, to confirm malaria infection, and, consequently, they treated presumptively. Therefore, although the information was understood, it did not encourage the necessary change in behaviour due to weaknesses in the health system.

On average, each workshop took 1 h from the objective assessment. By both objective and subjective assessment, the workshops were appropriately implemented (Table 4). The major objectively assessed weaknesses were: not recapping the previous or announcing upcoming topics, a lack of drama/songs to pull the crowd, and forgetting to self-report malaria cases since the previous workshop. During the objective assessment, it was also noted that most of the HAs were conversant with the topics. However, some of the weaknesses observed objectively included gaps in knowledge in a few HAs.

**Table 4:** Self-reported and MMP assessment for fidelity of the workshops

	Self-reported assessment	MMP assessment *n* = 4
% community workshops where the agenda is explained	100%	83%
% community workshops where new participants are introduced	98%	
% community workshops where the previous workshop is summarized	100%	58%
% community workshops where drama, song or a poem was done		42%
% community workshops where key points addressed	100%	75%
% community workshops where topic content is accurate		75%
% community workshops where lively discussion	100%	83%
% community workshops where self-reporting of malaria cases	100%	42%
% community workshops where next workshop’s topic is announced	97%	58%

Self-assessment data are pooled from 2015 to 2017. *MMP* Majete Malaria Project objective assessment were conducted in focal areas B and C in 2017

### Self-reported malaria cases and health services received

Self-reporting for malaria cases was used as a feedback for villagers to observe and evaluate changes in malaria burden within the community. Approximately half of the self-reported suspected cases of malaria were children. There has been little change in this fraction since 2015 (Table 5). The reported rate of rapid diagnostic tests (RDT) done remained between 85% and 87%suspected cases between 2015 and 2017. The reported RDT positivity rate declined from 82% in 2015 to 69% in 2016 and 72% in 2017, suggesting that there was an increase in treatment seeking for malaria-like signs and symptoms. Lumefantrine-artemether (LA), the first line malaria drug, was reportedly administered to more than 98% of RDT positive patients, suggesting that proper treatment was provided by the health system.

**Table 5:** Self-reported suspected malaria cases and health services received

Item	2015		2016		2017		*P*-value
	(*n* = 23)		(*n* = 26)		(*n* = 49)		
Suspected cases	350		351		567		
Under 5 (% of suspected cases)	189	(54%)	170	(48%)	299	(53%)	0.291
RDT done (% of suspected cases)	304	(87%)	299	(85%)	490	(86%)	**<** 0.797
RDT positive (% of RDT done)	248	(82%)	205	(69%)	353	(72%)	**< 0.001**
LA administered (% of RDT positive)	255	(103%)	211	(103%)	346	(98%)	

*n* is the number of workshops from which data were received. *P*-values of proportions across years were calculated from Chi-square statistic. Boldface *P*-value was statistically significant differences between the three years. *LA* lumefantrine-artemether; *RDT* rapid diagnostic test

## Discussion

Implementation of community workshops on malaria, as part of a broader development strategy, was clearly feasible in this rural setting. Trained community volunteers were able and willing to successfully implement community workshops for more than 2 years. The workshops were appropriate within the community as they received support from both village leaders and HSAs. Continued attendance of workshops by the community, with active participation and discussion, indicate that workshops were acceptable. The status received by HAs in the community motivated them to continue conducting the workshops. The HAs had accepted and taken up their role as change agents.

By introducing simplified malaria-related information in the local language, communities with a low level of education, many of whom were illiterate, had access to information on various aspects of malaria, including transmission, signs and symptoms, diagnosis, treatment, prevention and risk factors. The community was also involved by actively discussing how to apply this information to their daily lives. Such educational processes may be key to the effective use of interventions such as ITNs, prompt care-seeking for fever and intermittent preventive therapy (IPTp). Previous studies have shown that individual knowledge influenced the use of ITN [20, 21], uptake and adherence to ACT [22], and treatment seeking for fever [23]. There is also evidence that community engagement promotes the utilisation of ITNs in the longer term and increases the demand for bed nets [24, 25]. However, a barrier to understanding the information was attachment to traditional beliefs. There were preconceived ideas of malaria and its causes. These beliefs influenced how people behaved in relation to seeking treatment, similar to reports in other parts of Africa [26, 27]. The new information provided a different way of comprehending illness and promoted appropriate health behaviour.

As indicated in the interviews, there were challenges in maintaining a high attendance for the workshops as multiple factors influenced the attendance by the community. The successful implementation of the workshops relied heavily on the village leadership and their influence in the community. Villages where the chief and HSA were more involved had better participation of the workshops. The collaborative teaching with HSAs contributed to acceptability of the message in the workshops.

Attendance of workshops by both men and women was encouraging. Women were reported to most often note a child’s illness and therefore their attendance as primary care-givers was critical. For men, a previous study in the same district showed that men influenced the decision to seek care for febrile children [28]. Their attendance and participation in the village workshop therefore benefits the household if the information is accepted and used by both the men and women. Attendance by men in other areas was hindered by concurrent football matches. Football matches may be utilised as a platform to disseminate the health messages to the men. Understanding the community structure can provide more effective dissemination of information, and influence a desired change in health behaviour.

Our quantitative data indicated that the village workshops were implemented as required, with animators planning and hosting workshops with good attendance by the community. However, from the observations, some of the animators lacked knowledge on the topics. These were possibly the ones who replaced others and underwent a shortened orientation training, relying more on on-the-job training. They would have benefited from closer supervision and mentoring. Focus on observing and improving the quality of the workshops to maximise the outcome is needed [21].

According to the HAs, a direct benefit was a change in their own knowledge and behaviour through the understanding of malaria transmission, as they reportedly increased the use of ITNs and improved care-seeking for fever. This change in behaviour may have made them exemplary and credible in their communities. Responses from the HAs suggest that the approach of using the workshops as a platform to educate the community and influence a change in behaviour was both acceptable and appropriate.

The most notable behaviour change, according to the HAs, was prompt treatment seeking, which was not the norm prior to the implementation of the workshops. When there is community buy-in, with behaviour change taking place, the challenge may be unavailability of adequate interventions. There have been shortages in supply ITNs, malaria test kits and antimalarial drugs in the study area [29]. Lack of access to ITNs has been shown to limit the uptake of ITN in other studies [30]. There is a need to therefore strengthen the health system, to balance the demand for, and supply of services for the success, sustainability and effectiveness of such a programme; the supply of services would avoid people reverting to old habits [31].

The sustainability of the workshops after completion of the project is unknown. The workshops were conducted as a component of THP’s epicentre strategy in which an epicentre’s development is supposed to progress towards self-reliance, independent of project support. In this regard, it remains unanswered whether workshops are still needed after 2–3 annual cycles as a forum to address questions and problems related to malaria.

A separate survey to compare knowledge, attitude, and practice (KAP) between villages implementing the workshops and control villages is currently underway to measure these impacts. With an average of 10–17% of a village population participating per workshop, it would be important to study impact at village level. A cost-effectiveness analysis of implementing the workshops will also be conducted. Results from the current study, the KAP survey, and the cost-effectiveness analysis will be important for policy makers seeking to improve uptake of malaria control interventions.

The use of the community workshops is not limited to malaria control, but can potentially be used to address other health problems requiring behaviour change in a rural setting. The advantage of using this approach is that it benefits illiterate communities in a rural setting, which tend to have a high burden of disease. It is also appropriate and acceptable by both provider and beneficiary, which are key for uptake and local influential support.

## Conclusion

It is feasible to implement community workshops on malaria in a rural setting using local volunteers. Community workshops are appropriate and acceptable in rural communities, but require support from village leaders and health workers. The findings suggest that the community workshops are a potential tool for mobilising the community to improve their proactive role in malaria control, potentially changing their behaviour, and increasing their demand for health services. The community workshops can potentially be adapted for other health related problems, but need monitoring of their quality.

## Abbreviations

ACT: Artemisinin-combination therapy
AP: African parks
BCC: Behaviour change communication
CHW: Community health worker
COMBI: Communication-for- Behavioural- Impact
DHS: Demographic and Health Survey
FGD: Focus group discussions
HA: Health Animator
HSA: Health Surveillance Assistant
IDI: In-depth interview
IEC: Information education and communication
ITN: Insecticide-treated bed nets
KAP: Knowledge attitudes and beliefs
LA: Lumefantrine-artemether
MMIS: Malawi Malaria Indicator Survey
MMP: Majete Malaria Project
MWR: Majete Wildlife Reserve
RDT: Malaria rapid diagnostic test
THP: The Hunger Project
